# Low-Power Lossless Data Compression for Wireless Brain Electrophysiology

**DOI:** 10.3390/s22103676

**Published:** 2022-05-12

**Authors:** Aarón Cuevas-López, Elena Pérez-Montoyo, Víctor J. López-Madrona, Santiago Canals, David Moratal

**Affiliations:** 1 Universitat Politècnica de València, 46022 Valencia, Valencia, Spain; aaron@open-ephys.org; 2 Instituto de Neurociencias de Alicante, 03550 Sant Joan d’Alacant, Alicante, Spain; elena.perezm@umh.es (E.P.-M.); victor.lopez-madrona@univ-amu.fr (V.J.L.-M.); scanals@umh.es (S.C.)

**Keywords:** low power, data compression, wireless, brain, electrophysiology, FPGA

## Abstract

Wireless electrophysiology opens important possibilities for neuroscience, especially for recording brain activity in more natural contexts, where exploration and interaction are not restricted by the usual tethered devices. The limiting factor is transmission power and, by extension, battery life required for acquiring large amounts of neural electrophysiological data. We present a digital compression algorithm capable of reducing electrophysiological data to less than 65.5% of its original size without distorting the signals, which we tested in vivo in experimental animals. The algorithm is based on a combination of delta compression and Huffman codes with optimizations for neural signals, which allow it to run in small, low-power Field-Programmable Gate Arrays (FPGAs), requiring few hardware resources. With this algorithm, a hardware prototype was created for wireless data transmission using commercially available devices. The power required by the algorithm itself was less than 3 mW, negligible compared to the power saved by reducing the transmission bandwidth requirements. The compression algorithm and its implementation were designed to be device-agnostic. These developments can be used to create a variety of wired and wireless neural electrophysiology acquisition systems with low power and space requirements without the need for complex or expensive specialized hardware.

## 1. Introduction

Electrophysiology, recoding, and analysis of the electrical fields generated by the electrical cell activity remain one of the key tools used in neuroscience to investigate cognitive functions. Modern electrophysiology techniques allow the recording of large-scale cell populations, collecting both single-neuron activity dynamics as well as aggregated activity [1]. This results in signals which span a large range of frequencies, with useful information spread through all their spectrum. The nature of this data makes signal processing tasks a challenge.

Combined with appropriate behavioral tasks, electrophysiological recordings in freely moving animals have provided invaluable information to understand sensory processing, spatial navigation, memory formation, and decision making, to mention some examples. However, electrophysiological studies in behaving animals have been traditionally performed in well-controlled but severely constrained laboratory conditions, in relatively reduced size arenas or task apparatus, and involving a limited and often artificial (i.e., pressing a lever bar) repertoire of behaviors. Therefore, more natural and elaborated experimental conditions in ecologically meaningful contexts are required [2].

The need for open and meaningful spaces conflicts with the tethered nature of most electrophysiology systems, as it requires a physical connection between electrodes implanted in the experimental subject and the recording equipment. While this does not pose an issue for small, enclosed spaces and simple maze topologies [1,3,4,5,6,7], it prevents large arenas with enriched environments and social experiments with complex interactions with conspecifics (i.e., [8]). Wiring limits the distance the subjects are able to travel, can become tangled with environmental objects or damaged by the animals themselves. To solve these shortcomings, developments have been made towards wireless electrophysiology systems [9,10].

There are two main approaches to wireless acquisition: Data loggers and radio transmission. Dataloggers are devices running from batteries and able to store all recorded data into a local non-volatile storage medium. They have been used in a variety of animals, from fish [11] to birds [12]. However, the main drawbacks of this approach are its limited storage capacity and the impossibility of performing closed-loop experiments dependent on real-time data.

Radio transmission can transmit data to a remote receiver in real-time. Multiple methods exist for encoding and sending data through a radio stream. Analog neural data can be modulated, with multiple channels merged via time multiplexing and sent over a carrier frequency [13,14]. While an analog transmitter requires less energy [13], analog signals are more susceptible to noise than digital signaling, and the absence of an arbitration protocol prevents multiple devices from sharing the same frequencies.

Digital transmission can use simple one-directional carrier modulation [9], which alone adds noise resistance to the transmission or through complex protocols. Such protocols can add extra features such as synchronization, arbitration, or bidirectional control [15]. Some examples of widespread digital protocols are Bluetooth [16], a low power protocol designed for data rates up to 2 Mbit/s, Bluetooth Low-Energy [17], a slightly slower (up to 1.37 Mbit/s) version with reduced power needs or WiFi 802.11 b/g [18], a high-speed protocol with data rates of up to 54 Mbit/s and advanced arbitration capabilities, but with higher power requirements. Some projects have developed a custom protocol, being able to fine-tune the power-performance trade-off [19].

Power is the main bottleneck of wireless devices, limiting data rates and device operating life. Lowering power consumption allows for longer operational time, increased data rates, and reduced battery weights. As such, minimizing power requirements is a goal for every wireless device. In the case of radiofrequency systems, the power bottleneck stems from the power requirements of high-bandwidth data transmission [16]. Different approaches exist to reduce energy consumption in these devices. For example, developing the core hardware as a custom-made Application-Specific Integrated Circuit (ASIC) can help by integrating electronics highly optimized for the task [9,13] at the expense of increased development and production costs. Another line of improvement, since the bulk of power requirements stem from the radiofrequency transmission, is the development of specialized protocols which can yield improvements over generalist, commercial ones [20]. Research is also being made in fields like antenna optimization [21,22], to reduce the power needs of radiofrequency signals further, as well as optimize wireless power transmission.

A different approach, compatible with the previous ones and applicable to both data loggers and radiofrequency transmission, is to reduce the bandwidth needs of the data. A neural recording including fast activity transients, like spikes, require a sampling rate of at least 20 KS/s [23,24]. This combined with the multichannel acquisition, typical on modern high-density electrophysiology recordings, results in bandwidths of tens of megabits per second [22]. Compression techniques can be used to reduce bandwidth needs which, in turn, decreases the power consumption of the wireless transmitter.

A wide variety of compression methods can be used for neural signals [25]. One important parameter when considering a compression algorithm for a wireless implant is its complexity and power requirements. Some algorithms, such as wavelet compression, ref. [26] can yield excellent compression ratios but require circuitry capable of handling advanced mathematical operations. This creates two disadvantages: Extra computational needs often translate to extra power consumption, which diminishes the overall effect on power saving. In addition, these circuit requirements limit the number of devices in which they can be implemented. It is common that small, ultra-low-power commercial Integrated Circuits (ICs) do not have these advanced characteristics, making these algorithms only possible on higher-end devices.

In contrast, algorithms with lesser computational requirements, with lower compression ratios due to their simplicity, are often used on only a particular part of the signal spectrum. For example, lower-frequency Local Field Potentials (LFP) tends to have high inter-channel redundancy, making high compression ratios with simple techniques possible [27]. High-frequency spikes, in contrast, are sparse events, so it is possible to use spike-detection algorithms and only perform compression for the discrete, individual events [28,29,30,31]. Both techniques can be combined, compressing and sending both LFPs and spikes separately by the same device [32,33]. These approaches, however, are not able to provide a complete, continuous view of the entire acquired signal.

A fundamental characteristic of compression algorithms is the accuracy of the reconstruction of the original signal after it has been compressed. In this sense, lossless algorithms produce, after decompression, signals identical to the original ones, while lossy algorithms, which generally have a higher compression performance, introduce, however, signal distortions [25,34].

An example of a low-resource lossy compression algorithm is compressed sensing [35,36]. This method works by sampling a signal below the Nyquist frequency, thus reducing data size [37] with negligible requirements on power or resources in the encoder. The computational burden lies entirely in the decoder, which must reconstruct the signal through complex mathematical operations [35,36,38,39]. This method is, however, a lossy algorithm that introduces distortions in the data. Moreover, both its compression efficiency and signal distortion are affected by acquisition noise [40]. While the simplicity of the encoder makes it a good candidate for wireless devices [31,33,41], it is limited by the distortions it introduces.

Here we describe a lossless compression algorithm for brain electrophysiology, able to reduce bandwidth and, by extension, transmission power requirements, along with a novel hardware implementation focused on resource minimization. It can compress data to 40–60% of its original size with no signal distortion and requires little power for its processing. This algorithm is based on a combination of delta compression and Huffman coding, both requiring little computational power and thus adding minimal extra power needs. The algorithm implementation is optimized to minimize hardware resources, not requiring any specialized hardware, which makes it possible to be used in a wide range of devices including low-cost or small ultra-low-power ICs. This, coupled with its ability to be configured for any number of channels and sampling rates, offers great flexibility for designing a variety of battery-powered wireless acquisition devices suitable for different experimental needs.

**Figure 1:**
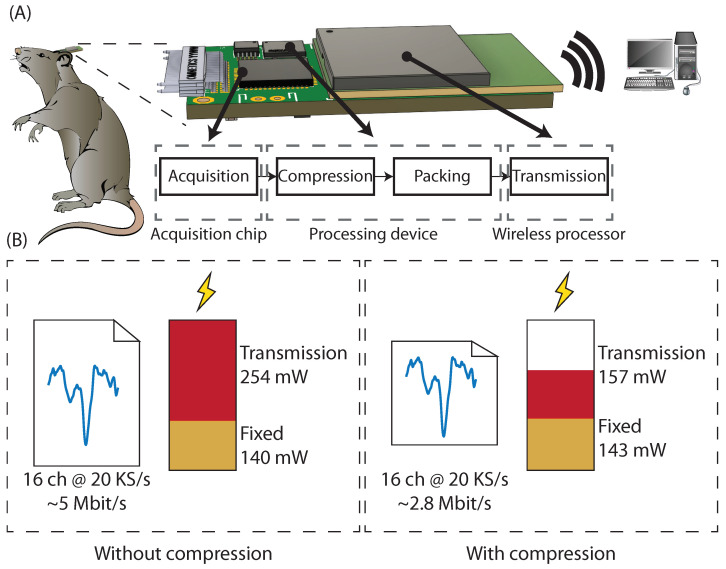
(**A**) General overview of the system and the built prototype. (**B**) Differences of power and bandwidth requirements of the prototype with and without enabling compression.

In addition to the algorithm, a low-overhead communications protocol was designed to allow compressed data to be efficiently shared between components for cases in which the electronic device for compression is different from the wireless transmitter or storage controller. Finally, a hardware prototype was created, implementing all the designs, and tested in vivo to validate the compression ratios and the resulting power consumption reduction in transmission, which decreased in a similar proportion as the bandwidth. Figure 1 shows an overview of the developed systems and devices.

## 2. Materials and Methods

### 2.1. Huffman Coding

Huffman coding is a method to encode information devised in 1952 by David Huffman [42]. The basic precept behind Huffman coding is that, in any set of symbols, the appearance frequency of every different symbol might not be the same. Under that case, a usual fixed-length coding is highly redundant, as defined by Shannon’s theorems, [43] and, thus, not optimal. Huffman coding, instead, codes each symbol with a different bit length, depending on their appearance frequency. This way, symbols that appear more frequently are coded with fewer bits than less frequent symbols, reducing the overall bit size of the set. A natural consequence of this encoding is that compression rates are higher when symbols follow a steep distribution, i.e., a few subset symbols conform to the majority of the set, while flat symbol distributions result in low compression rates.

To use Huffman coding, a specific dictionary must be created for each different data set, analyzing symbol appearance frequency and generating the optimal codes for each [44]. This implies prior knowledge of the data to be compressed. For real-time applications, the dictionary can be made with previously recorded data, with the optimal compression ratio becoming a mean ratio. For this method to be accurate, the prerecorded set has to be large enough so its symbol frequency appearance matches the symbol appearance probability on real-time data.

Due to the variable nature of Huffman coding, dictionary size can vary depending on the datasets and the resulting codes, with a maximum possible size of $2^{2n}$ bits, for a collection of *n* bits symbols [45]. Different algorithms exist to reduce this size. Some force a maximum code length [46] which reduces the algorithm efficiency. Others work by rearranging the dictionary after it has been created [45], which requires it to be known in advance to measure the memory needed.

**Figure 2:**
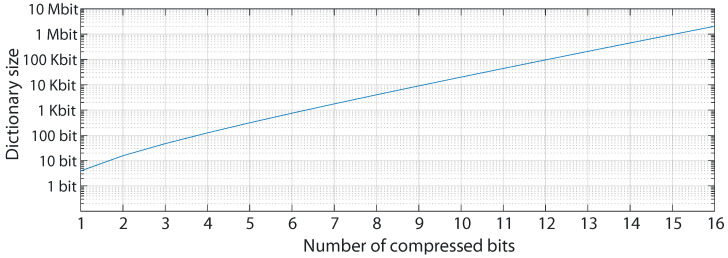
Dictionary size needed by the selected variation of the Huffman algorithm [47] for datasets of word lengths of 1 to 16 bit.

We have built an algorithm based on [47] that minimizes dictionary size, being solely dependent on the bit word with the symbols in the dataset, and without limiting code length. In this algorithm, a collection of *n* bit symbols requires $2^{n+1}$ bits of memory, as shown in Figure 2.

### 2.2. Delta Compression

Delta compression, or delta encoding, is a very simple method in which each symbol is represented with the difference with the preceding one, i.e., $y_{i}=x_{i}-x_{i-1}$. Decoding can be done by cumulative addition of the received values $x_{i}=x_{i-1}+y_{i}=\sum_{n=0}^{i}y_{n}$.

This coding is especially suitable for signals that follow a smooth progression, with high-frequency components having a low amplitude, such as those of biological origin [48]. For signals with these characteristics, the resulting difference vector y=Δx is composed of a majority of low values, which can be encoded with fewer bits instead of the more even symbol distribution that the raw signals have.

### 2.3. Hardware Prototype

**Figure 3:**
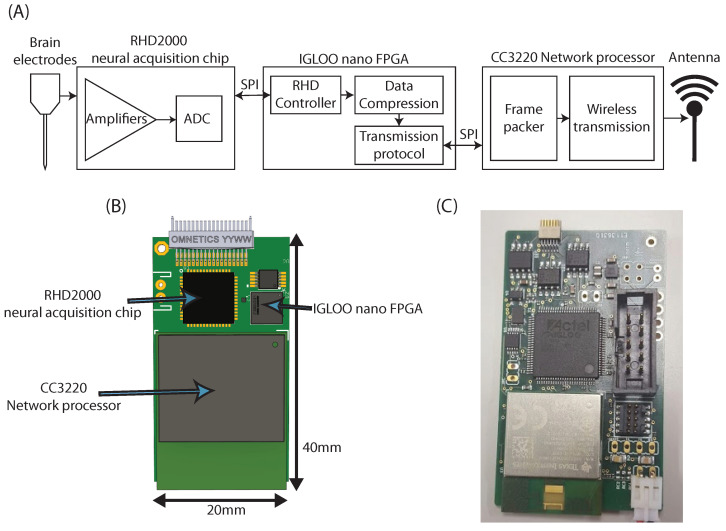
(**A**) Functional diagram of the developed prototype. (**B**) Sketch of a complete device. (**C**) Picture of the built prototype. For development purposes, the layout differs from what would be a finished unit, including the addition of debugging headers and the use of bigger versions of the integrated circuits.

Although the main purpose of this work was to create a device-agnostic compression and transmission scheme that could be used in any ultra-low power device, a complete system was designed for testing. It led to producing a fully functional prototype implementing acquisition, compression, and wireless transmission. Figure 3 shows a functional diagram of the prototype as well as a picture of the built device.

#### 2.3.1. Low-Power FPGA

The central device of the prototype, driving neural acquisition and compression, is an ultra-low-power FPGA. The chosen device is an AGLN250 IGLOO nano FPGA (Microsemi, Alto Viejo, CA, USA) [49], featuring both a small footprint and low power operation. The drawback of this kind of device is its reduced resources, with no hardware multipliers or Digital Signal Processor (DSP) modules and little available Random Access Memory (RAM). This showcases the importance of the low-resource approach taken when designing the algorithms. IP cores were developed in the Verilog language to implement the design.

#### 2.3.2. Acquisition Chip

The device selected for the neural acquisition was an RHD2132 chip (Intan Technologies, Los Angeles, CA, USA) [50]. This integrated circuit can acquire and digitize up to 32 channels at 30 KS/s, having a 16-bit output with a resolution of 0.195 μV per bit and 2.4 $\mu V_{\mathrm{rms}}$ input noise. Communication with the driver device is done through an SPI bus, a standard communications protocol in electronics.

#### 2.3.3. Wireless Device

Wireless communication in the hardware prototype was performed through the WiFi IEEE 802.11g protocol. Although several studies have demonstrated how custom protocols can offer very efficient wireless transmission, ref. [19,20] the use of a standard, widely available protocol allows for an easy way of testing the efficiency of the compression algorithm implementation independently of any transmission-related factors. While other off-the-shelf devices exist designed for low-power transmission, they often offer low data bandwidths. While the objective of this work is to compress neural data so it can fit such devices, using a higher-bandwidth device allows the design to be characterized without external bandwidth constraints. Moreover, the wide availability of commercial devices for both transmission and reception, as well as the interference protection features of the protocol, makes it a perfect candidate for testing-phase prototype building.

The major downside of the protocol in the context of this work is that it is not natively designed for low-power applications. However, commercial devices exist that reduce the power requirements to a minimum and can operate on batteries. While the 802.11g protocol has little provision for reducing its power levels in relation to the required bandwidth, these devices can send data in bursts at full speed and power down the transmitter circuitry when not in use. This means that a reduced data rate, as achieved by compression, still translates as lower power usage even with a non-optimal protocol.

A transceiver device with an integrated network processor was used, specifically the CC3320SF IC (Texas Instruments, Dallas, TX, USA) [51].

#### 2.3.4. Development Hardware

For early development and testing stages, the Verilog designs were implemented on the Open Ephys acquisition hardware (Open Ephys, Cambridge, MA, USA) [52], which features a mid-range Spartan-6 FPGA (Xilinx, San Jose, CA, USA) and the same RHD2132 chip. This allowed us to verify the compression algorithm with in vivo experiments before building the wireless prototype. A plugin for the Open Ephys software was developed to receive and decompress the signals provided by both the implementation on the Open Ephys hardware and the wireless prototype.

### 2.4. Sample Signals

The compression algorithm implementation was designed to be optimized, both in performance and resource efficiency, for brain electrophysiology signals. The different development and design stages required the use of sample data sets of electrophysiological recordings. In particular, 10-min recordings from the hippocampus of two different rats and a 5-min recording from the visual cortex of a third rat were provided by the Alicante Neuroscience Institute (San Jose de Alicante, Alicante, Spain) and the Open Ephys project (Cambridge, MA, USA), respectively. In both cases, the data was acquired using the Open Ephys hardware.

These sample datasets were used for all measures and decisions leading to the low-resource design presented. The Huffman dictionary used throughout the design was created from the combination of these signals after being processed by the modified algorithm developed in this work.

To test algorithm performance on signals not related to dictionary creation, data from two sets of animals were used. Five-minute recordings from the retrosplenial cortex of five different mice were provided by Jakob Voigts, from the Harnett lab, at the Massachusetts Institute of Technology (Cambridge, MA, USA). Canals Lab at Alicante Neuroscience Institute (San Juan de Alicante, Alicante, Spain) provided data from an independent experiment involving three Long-Evans rats implanted in the hippocampal region. From these animals, 30 min of data were recorded daily for four consecutive days. Both these datasets were processed offline by the algorithm to measure compression ratios.

An in vivo experiment was also performed with three rats in the Alicante Neuroscience Institute to test the algorithm implementation with online compression. Data from these animals was recorded with a modified version of the Open Ephys hardware integrating the complete compression algorithm in its final low-resource, low-power implementation, obtaining 20-min recordings with data compressed in real-time.

## 3. Development and Design

### 3.1. Compression Algorithm

Neural compression is achieved in this work by the combination of both delta and Huffman encoding. This combination creates a lossless algorithm that can be implemented with simple hardware, requiring only a small amount of Read-Only Memory (ROM) and simple logic gates. This enables its implementation in simple devices with few resources, such as ultra-low power FPGAs. The complexity of the algorithm is dictated by dictionary size, which, as seen in Figure 2, is directly related to the number of bits on the samples to be compressed. While execution complexity is O($log_{2}\left(n\right)$), storage requirements are O($2^{n}$), related to the bit width of the signal. Thus, a significant part of this work is directed toward reducing storage requirements in the specific case of neural signals.

**Figure 4:**
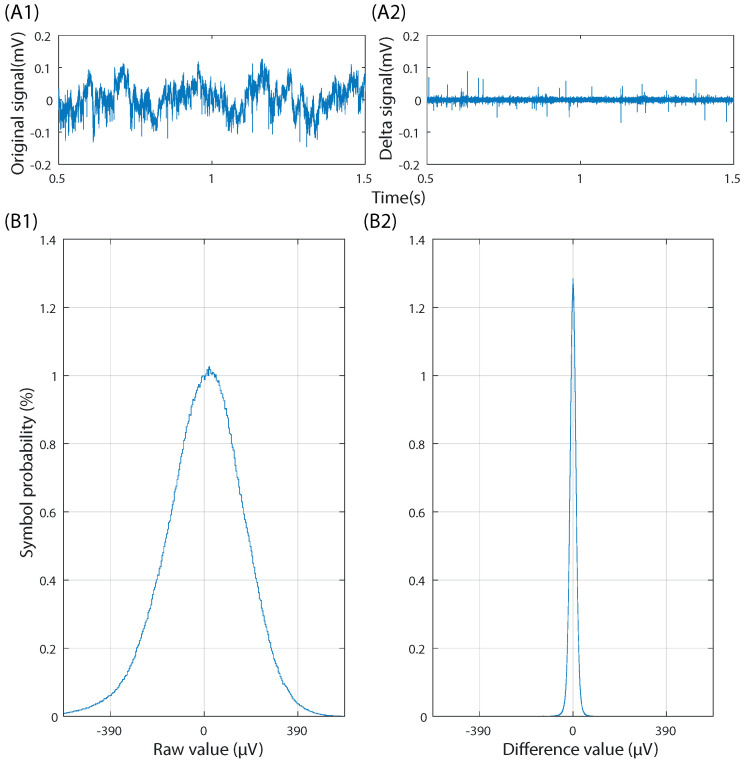
Comparison between a 20 min neural signal in raw form (**A1**,**B1**) and after delta-encoding (**A2**,**B2**). (**A**) 1 second time-domain sample of the raw signal (**A1**) and the delta-encoded signal (**A2**). (**B**) Symbol probability of the full recording in raw form (**B1**) and after delta-encoding (**B2**).

While neural signals are not optimal for Huffman compression in raw form, this can be improved by the derivative transformation caused by delta encoding. Figure 4 shows how delta compression can optimize electrophysiological recordings for its use with Huffman encoding. It can be seen how, after performing delta compression, the distribution becomes steep, with low-value symbols being orders of magnitude more frequent than higher-value ones. Thus, by using delta encoding on a neural signal, it becomes optimal for further compression using the Huffman method. The Huffman dictionary is thus elaborated from delta-compressed neural signals. We treat each channel of the multichannel recording separately to achieve the maximum possible compression while being tolerant to as many electrode configurations as possible.

### 3.2. Low-Memory, Low Resource Compression

While neither delta compression nor Huffman coding requires specialized Digital Signal Processor (DSP) or multiplier circuitry, which would limit the range of low-power devices they could be implemented on, Huffman coding requires a ROM memory containing the symbol dictionary. The algorithm version used in this work is already designed to minimize memory needs [47]. However, as seen in Figure 2, for 16-bit words, which are typical in the neural acquisition [23], this results in 2 Mbit of memory, limiting the available devices able to run this algorithm. A number of ways were devised to reduce the word width and thus the dictionary size while minimizing the impact on compression ratio and signal integrity.

**Figure 5:**
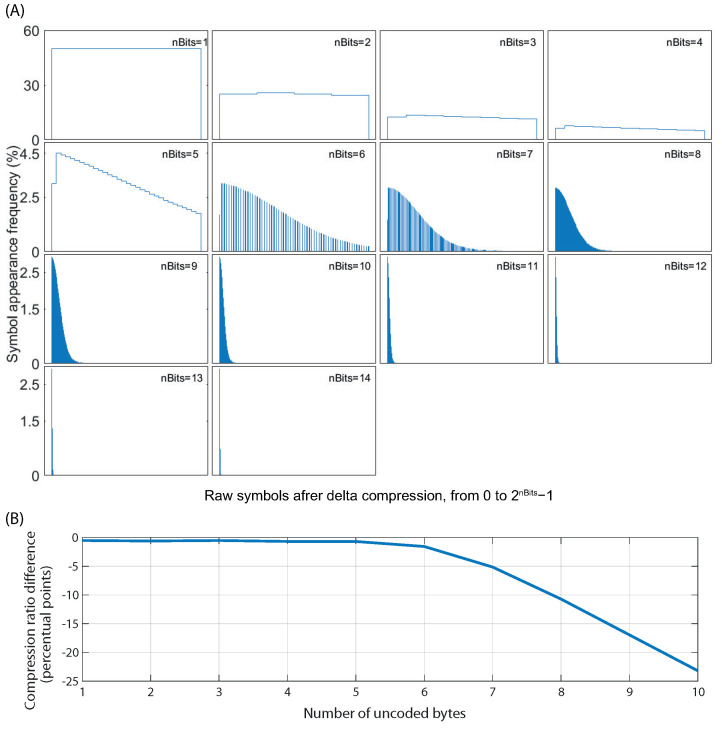
(**A**) Symbol distribution of a delta-coded sample signal, in absolute value, for a different amount of masked bits. The lower nBits are kept and the others discarded before plotting the probability distributions. The X-axis of each plot are the different symbols, from 0 to $2^{\mathrm{nBits}}$. (**B**) Degradation, in percentual points, of the compression efficiency when the different amount of bits are transmitted without being coded by the Huffman algorithm.

Delta encoding, being a binary subtraction, already trims one bit, leaving 15-bit words to be compressed. To further reduce the number of bits needed by the Huffman dictionaries, not all bits are coded using that process. The efficiency of the Huffman algorithm relies on the appearance probability of a small subset of symbols being higher than the rest. However, as evidenced by Table 1, not all bits of a delta-compressed signal follow the same distribution, with only higher bits contributing to the steepness of the distribution, as shown in Figure 5A.

It is possible then to only use Huffman encoding on the higher bits while appending the lower bits of the delta-compressed signal without further processing. Figure 5B shows how compression efficiency is affected by this approach. Moreover, the probability distribution is symmetrical, which allows one to create a Huffman dictionary for only absolute values and appending the sign bit unprocessed as well.

Two extra steps are being incorporated into the algorithm to improve the compression ratio even further. As the sign bit has only relevance for nonzero values, it is not transmitted when the decoded value is zero. Additionally, although analog to digital converters (ADC) circuits usually have 16 bit outputs, the conversion process produces a lower number of relevant bits, with the less significant bits being electrical noise. Those can safely be omitted, as they contain no useful data by design, further reducing bandwidth needs. In the case of the device used in this work, with 0.195 μV resolution and 2.4 $\mu V_{\mathrm{rms}}$, it is possible to calculate that the output contains $log_{2}\left(2.4/0.195\right)=3.6$ bits of noise. Thus, the three lower bits can be completely discarded instead of being sent uncompressed reducing the actual bit width of the signals to 13 bits. This is a conservative amount to ensure trimmed data is below the noise floor of the amplifier. For other acquisition devices, the number of discarded bits can be adjusted so they are always below the input noise level, resulting in small differences in compression ratio.

**Figure 6:**
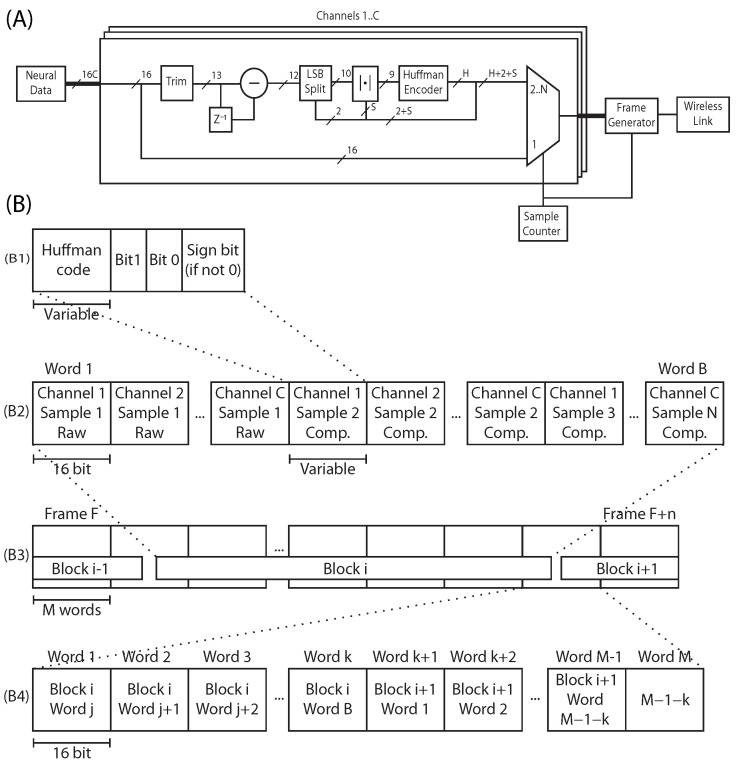
(**A**) Block diagram of the complete system, detailing the compression algorithm. H stands for the variable bit count of a Huffman-coded word, while S can be 1 bit for sign coding, or 0 bits for 0-value words. (**B**) Structure of the different protocols. (**B1**): Compressed sample. (**B2**): Compressed block of N samples for C channels. Total size of B word can vary depending on compression. (**B3**): A block spans several frames, while a single frame can include the boundary between two blocks, (**B4**): Frame of M words sent to the transmitter, with index to detect block boundaries.

A complete block diagram deletedof the algorithm can be seenvisible in Figure 6A. The Huffman dictionary needed for the algorithm is, thus, made from the sample dataset after it has been treated by this process, considering only the bits that are to be compressed by the Huffman method.

### 3.3. Transmission Protocol

Devices for neural compression and wireless transmission data might not be the same. In addition, many low-power wireless protocols lack mechanisms to ensure reception, thus being susceptible to packet loss [18]. This is especially problematic for delta coding as each lost incremental value introduces a permanent error in the signal, which increases with each consecutive missed value. A protocol was designed to transmit data between devices, including information enabling the wireless processor to pack the data in a way able to recover from packet losses. This protocol utilizes few hardware resources, has no RAM requirements, and adds a low overhead to the transmission, maintaining the reduced bitrate achieved by the compression.

Data is packed in blocks of N samples, with the first sample for each channel being the raw, uncompressed values, followed by the remaining compressed samples. If the network packet aligns with block boundaries, in the case of a packet loss, the receiver could recover at the start of the next block. Huffman coding introduces an additional challenge due to the resulting samples having a variable bit length. The network processor must track block boundaries independently of their actual byte size.

Data from the compressing device to the wireless processor is packed in fixed-length frames of M words, with the current implementation using 16-bit words. The first M-1 words are comprised of block data. The last word is a block boundary indicator. If the frame contains the start of a new block, the indicator is an index pointing to the specific word of the frame in which the new block starts. If the whole frame contains data from the same block, the indicator is a value greater than M. This transmission protocol allows the wireless processor to be fully aware of delta-coded block boundaries without requiring the compressor device to store them in memory. With this information, the wireless protocol can add simple indexed markers to the data packets, allowing the receiver to detect a packet loss and wait for the next delta-coded block to start.

Figure 6B shows the frame and block structures and the byte composition of a compressed sample.

## 4. Results

### 4.1. Compression Performance

As a Huffman dictionary provides the optimal compression ratios for the data used to create it, algorithm performance in real experimental situations was measured using datasets not related to dictionary creation. From the 385 min of data from eight different animals available for offline compression through a software model of the algorithm, an average ratio of 47.94% of the original signal size was achieved (Table 2).

In vivo real-time compression, using the hardware implementation of the algorithm, yielded a mean ratio of 65.58%. It is worth noting that one of the three experimental animals, which will be called Rat 3 from now on, they had an uncommonly high amount of acquisition artifacts. As a result, the performance of the algorithm was slightly affected. Removing the data from this animal results in a mean compression ratio of 62.64%.

Table 2 shows the detailed ratios for each of the animals and setups.

### 4.2. Signal Integrity

The combination of delta compression and Huffman coding in their original forms is completely lossless, introducing no alteration to the input signal in the process of compression and decompression. In the implementation described in this work, we alter the input signal by removing the trailing bits of the input signals, corresponding to the input noise of the acquisition circuit.

**Figure 7:**
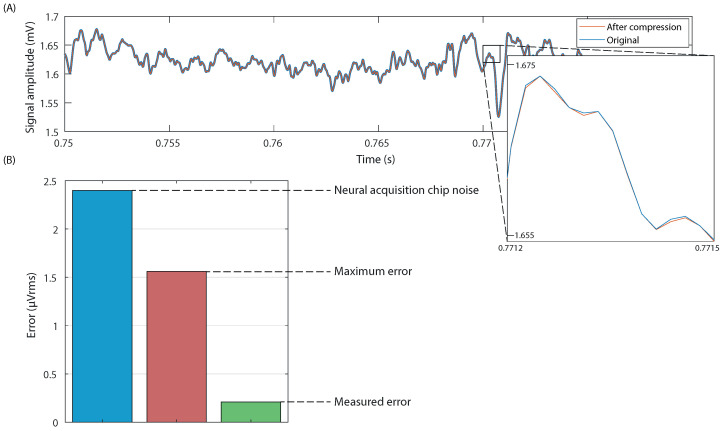
Effect of compression on signal integrity. (**A**) Compressed and original signals. The error is indistinguishable without magnification. (**B**) Error introduced by the algorithm compared with the acquisition chip noise floor.

However, the only effect this procedure has on signal integrity is the introduction of noise below the noise floor of the acquisition chip itself, thus not affecting the actual acquired data. Figure 7 show a comparison between an original and a processed signal. The measured error is 0.21 $\mu V_{\mathrm{rms}}$, while the maximum possible error introduced by the current implementation of the compression algorithm is 1.56 $\mu V_{\mathrm{rms}}$, all below the 2.4 $\mu V_{\mathrm{rms}}$ noise of the neural acquisition chip itself.

To further evaluate the effect, the mice datasets were processed by an automatic spike-sorting algorithm [53] both in their raw form and after being compressed and decompressed, with results shown in Table 3. There was a mean event match of 99.98%, with the remaining 0.02% being not spikes but noise-related events close to the detection threshold. On the spikes, the specific sample that triggered the event matched with an error of 0.308 samples. Clustering showed identical sets in both cases.

### 4.3. Effect of Dictionary on Compression

As the Huffman dictionary was created with a sample set of signals, it was of interest to ascertain whether creating dictionaries using some datasets of the same animals in the experiment provided any performance variation. This was tested using the 4-day dataset. Dictionaries were made from the data acquired during the first day. The whole set was then compressed offline using the base dictionary, the dictionary made from each of the rats, and combinations of these dictionaries with the original.

Table 4 shows the obtained compression ratio for different combinations. A slight improvement from the base results can be observed when using dictionaries, including data from the experimental dataset. As expected, removing the data noisy from Rat 3 improves the results.

### 4.4. Power Usage

Bandwidth reduction, which compression achieves, can reduce power in two main ways: by allowing the usage of low-power protocols, which often have a lower bandwidth associated, or by enabling a higher bandwidth protocol in small bursts, increasing the time the device is not transmitting. In the case of the CC3220 network device used, it can be configured, so the wireless circuitry enters a lower-power state between operations. This way, fewer data to transmit translates to smaller bursts and longer sleep times for the wireless circuitry, thus reducing transmission power accordingly.

To measure power usage on the prototype, test points were added to independently measure the current consumption of the FPGA, wireless processor, and acquisition device. Accurately measuring the specific effect of compression required a known and noise-free signal to be transmitted both raw and compressed, and transmission power to be measured in both. To this avail, a 16-channel, 20 KS/s synthetic signals, made using simple arithmetic progressions but mimicking the post-delta mean symbol distribution, was used. The signal was generated inside the FPGA and transmitted to the network processor in both compressed form and raw, bypassing the compression algorithm to compare power usage between the two cases.

**Figure 8:**
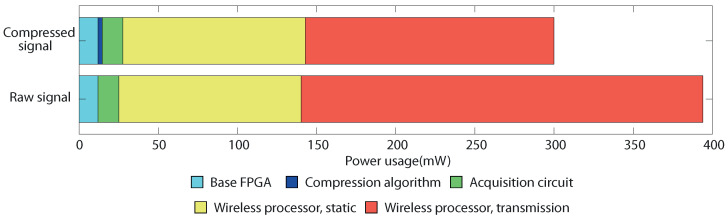
Power usage of the sample hardware implementation transmitting the compressed signal and the raw, uncompressed signal.

Figure 8 shows the power usage of the wireless prototype. It can be seen how the amount of extra power used by the compression algorithm, measured at 2.7 mW, is negligible. As the Wi-Fi protocol is not designed specifically for low power, it features a high, static consumption dedicated to maintaining the link, even when it is not transmitting. However, even in this non-optimal case, the measurements demonstrate a clear reduction in transmission power, directly related to the decrement of required bandwidth.

### 4.5. Resource Usage

Minimizing hardware resources was an important objective, as this allows the algorithm to be used in a wider variety of existing devices and makes it more efficient to be integrated into an ASIC. This includes both memory and logical requirements. In the case of the former data size of the Huffman dictionary was reduced. Instead of the 2 Mbit a 16-bit dataset would need, only 9 Kbit were required. FPGA logical resource usage was kept minimal and no DSP blocks or any other specialized hardware modules were required. Table 5 shows the FPGA cell usage of the different modules for both Xilinx and IGLOO nano FPGAs, as well as the percentage of the device used in the prototype.

## 5. Discussion

Studying complex and ecologically meaningful behaviors in animals is necessary to move experimental cognitive neuroscience forward [54], but requires experimental conditions closer to the natural conditions or even experiments in the real world. This often implies large spaces filled with elements such as obstacles, hiding places or even burrows, and environments shared by multiple animals. All these elements render devices tethered to the animals impractical, as the wiring would limit mobility and animal-animal or animal-context interactions.

Wireless implants can record brain activity during extended periods of time allow free movement of animals in complex environments, opening the possibility to a new generation of neurophysiological investigations in behaving animals.

For a wireless device, autonomy is crucial, with power usage being often the most limiting factor. Wireless data transmission has large power requirements, depending on the data rate, with higher rates requiring faster and more powerful signal processing. Reducing data rate lowers the power needs by either using slower, less power-demanding protocols or allowing the transmitter to be in a powered-up state for only brief periods of time, sending small bursts, and keeping it in a powered-down, low-power state most of the time.

Compression is an efficient technique to reduce the data rate, but only if the power needed for compression is lower than the power saved by rate reduction. However, some compression methods can distort the integrity of the data. A lossless compression system for brain electrophysiology must be able to faithfully transmit all the information contained in the wide range of the signals, which spans from 0 Hz to several kHz. This is the case for the compression algorithm presented here, which has demonstrated both its low energy footprint and power reduction during wireless transmission. This reduction was demonstrated on a regular Wi-Fi IEEE 802.11g chip. While useful for testing, this device is designed for high-bandwidth and not optimized for low power, with high energy consumption in static link usage. Using custom wireless protocols or specialized low-power devices, will reduce transmission power needs, further decreasing power needs. Especially interesting are the recent developments on IoT-related wireless protocols and devices, such as IEEE 802.11AH [55], designed for low-power transmission while allowing a variety of different data rates.

Although this compression scheme was originally designed for wireless transmission, it could easily be adapted for other electrophysiology applications. Data loggers are an immediate example, as the algorithm would add negligible extra power and resource requirements while doubling the capacity of storage devices, thus greatly increasing the system autonomy. Wired acquisition systems can also benefit from compression, as link bandwidth often limits the maximum possible channel count in headstages. An example of such an ultra-high channel count system that could benefit from the ability to integrate more probes per headstage would be Neuropixels, high-density CMOS-based neural probes [56,57].

This flexibility of usage is reinforced by the low-resource nature of the development. Being kept with minimal hardware needs makes the algorithm easy to fit in existing designs, being able to be implemented in a variety of devices. This is also important for power consumption as, unless highly-optimized custom chips are used, devices with more hardware resources tend to be bigger and with more power requirements. Being low-resources makes it possible to be implemented in simple, low-power, commercial chips.

The focus on implementability in a diverse range of low-power, commercially available devices, including low-end ones, imposes hard limits on the algorithm complexity and, by extension, performance. Similar algorithms focusing on lossless or near-lossless compression can achieve ratios of near 20% [58] by separating LFP and single spikes and compressing them independently. This has one downside of defining a hard frequency threshold, with the risk of losing data in the middle range. Moreover, band separation requires the use of digital filter circuitry, which might not be present on all commercial devices. As a counterpart, similar results to ours of approximately 50% reduction can be achieved by exploiting spatial redundancy [59]. Although this approach requires some extra resources, which are more easily adjusted in a custom-made ASIC, it could also be used in many existing hardware. Coupling both algorithms could yield increased results by exploiting both temporal and spatial characteristics. As a comparison, lossy algorithms can achieve data reductions below 10% of the original size [41] by introducing distortion to the neural signals or focusing on specific parts, such as compressing and transmitting Spikes only [39].

The developed transmission protocol further reinforces the flexibility of the algorithm and its implementation by being able to maintain long-term signal integrity in the cases where data losses are expected. This might be the case for ultra-low-power wireless transmission protocols, as the drawback of expending less energy on link maintenance is the possibility of short interruptions on transmission, with their related packet losses. Being able to recover from such events makes the complete design suitable for almost any situation.

Data integrity and compression efficiency are two elements that must always be balanced. In this work, the compression algorithm was developed with the former in mind, being virtually lossless, with compression noise being below the noise floor of the acquisition chip. There are methods in which the compression ratio can be increased while introducing noise into the signal. One such way is in the delta coding step. As seen in Figure 4(B2), large delta values are rare and often the result of acquisition artifacts. Those uncommon, large values could be removed by trimming the most significant bits, further reducing word width [60]. In this case, any time such a large jump occurred, either naturally or by an acquisition artifact, the DC offset of the signal would drift from its real value while maintaining most of its characteristics. In this case, the signal would be corrected at the start of the following block. Another way to increase compression would be to trim even more bits before delta coding. This would result in a loss of resolution, with an equivalent noise of $V_{\mathrm{LSB}}*2^{\mathrm{nRemovedBits}}$. Conversely, if an acquisition chip with a lower noise floor were used, the number of discarded bits could be lowered, albeit with a slight impact on compression ratios.

Compression efficiency can also be improved without degrading signal quality by the optimization of the Huffman dictionary. Section 4.3 shows how creating a customized dictionary with data previously recorded from the same experimental animals can increase compression. Understanding the specific factors that lead to these improvements could help further improve the performance. Current suspicions point to them being related to the physical properties of the experiment, such as electrode impedance and acquisition rate, which affect how the signal varies over time and such the result of delta coding. More research on this topic needs to be done to optimize further the procedure presented here.

## 6. Conclusions

A low power compression algorithm for brain electrophysiology signals combining delta compression and an optimized implementation of Huffman coding was developed. This algorithm can compress neural data to nearly half its original size in a lossless manner without adding any distortion. Compression efficiency can be slightly improved by customizing the dictionaries using data from the same experimental animals.

This algorithm uses minimal hardware resources, making it possible to be implemented in low-power devices. A protocol for packing the compressed signals with little overhead and the capability to recover from packet losses was also developed for its use with wireless transmission. The compression algorithm and the transmission protocol add negligible extra power usage to the system, favoring the implementation of the algorithm in a variety of wireless electrophysiology acquisition systems.

Reducing bandwidth naturally reduces the power needed for a wireless transmission protocol. This was verified in a prototype wireless acquisition system created using commercially available, low resource, and low-footprint devices. Although the transmission protocol utilized in this work was not designed for low power, a sizable reduction in power consumption was achieved due to data compression. 

## Figures and Tables

**Table 1:** Frequency of each bit of a delta-coded sample signal having a value of ‘0’ or ‘1’ when taken as an absolute value. Bits 9 to 14 are not shown as their probability being ‘1’ is exponentially reduced each step. For the Signal bit, only nonzero values were counted, with ‘0’ meaning a positive signal and ‘1’ negative.

Bit	0	1
0	50%	50%
1	50.72%	49.28%
2	52.19%	47.81%
3	55.15%	44.85%
4	61.36%	38.64%
5	77.18%	22.82%
6	95.51%	3.49%
7	99.81%	0.19%
8	99.99%	0.01%
9	∼100%	<0.01%
Sign	49.76%	50.24%

**Table 2:** Compression ratios, in the percentage of the original signal size, for the different datasets not related to dictionary creation.

Data	Compression Ratio	Recording Time
Offline compression		
Mouse 1	33.86%	5 min
Mouse 2	33.72%	5 min
Mouse 3	33.37%	5 min
Mouse 4	33.25%	5 min
Mouse 5	38.44%	5 min
Rat 1	51.37%	30 min × 4 days
Rat 2	44.60%	30 min × 4 days
Rat 3	59.31%	30 min × 4 days
Mean	47.94%	Weighted Average
in vivo online compression		
Rat 1	62.99%	20 min
Rat 2	62.29%	20 min
Rat 3	71.45%	20 min
Mean	65.58%	Average

**Table 3:** Events were detected by spike sorting software in raw and processed signals from five different mice.

	Mouse 1	Mouse 2	Mouse 3	Mouse 4	Mouse 5
Detected events in original	11,021	139,743	109,759	207,416	148,400
Detected events in processed	109,992	139,709	109,763	207,379	148,374
Matching events (%)	99.9736	99.9757	99.9964	99.9822	99.9825
MAtching events start sample error	0.3572	0.3910	0.3404	0.2755	0.1437

**Table 4:** Mean sizes of the compressed signals relative to the original data for in vivo tests. Columns for dictionaries using experimental data alone or added to the base dictionary. Rows for base dictionaries, dictionary from data from all the animals (All), dictionaries from data from each individual rat (Self), or dictionaries from data from all animals except the one being tested (Others). Data shown including and excluding the anomalous rat labeled “Rat 3”.

		Animal Data	Animal + Base
Base	w. Rat 3	51.87%	N/A
	w/o Rat 3	48.15%	N/A
All	w. Rat 3	50.76%	50.59%
	w/o Rat 3	47.42%	47.96%
Self	w. Rat 3	48.8%	49.42%
	w/o Rat 3	46.42%	47.44%
Others	w. Rat 3	49.37%	51.16%
	w/o Rat 3	47.67%	46.39%

**Table 5:** Prototype usage percentage measured for the Microsemi AGLN250 device.

	Xilinx Cells	IGLOO Cells	Prototype Usage
Compression	60	585	9.20%
Transmission protocol	22	210	3.42%
Data acquisition	54	495	8.08%

## Data Availability

Not applicable.

## Abbreviations

ASIC	Application-Specific Integrated Circuit
DSP	Digital Signal Processor
FPGA	Field-Programmable Gate Array
IC	Integrated Circuit
LFP	Local Field Potentials
RAM	Random Access Memory
ROM	Read-Only Memory
